# Mechanical Dentistry

**Published:** 1865

**Authors:** Geo. E. Hayes


THE
### DENTAL REGISTER OF THE WEST.

Vol. XIX.] MARCH & APRIL, 1865. [Nos. 3, 4.

### Original Essays and Communications.

### MECHANICAL DENTISTRY.

**Read before the Buffalo Dental Association.**

BY GEO. E. HAYES, D. D. S.

Gentlemen: You have asked me to write on the subject of Mechanical Dentistry. Before responding to this request, it may be well to enquire, what is meant, precisely, by this phrase. Being of Yankee origin, I will answer by proposing another query, what kind of dentistry is not mechanical? If a patient calls with the tooth-ache, you go at once to the tool case. If he only fears that malady in the future, you handle the probe. If you find carious teeth which you judge may be rendered useful in the future, your mechanical faculties are called into exercise. You select a material which mechanically will exclude moisture, and protect the tooth from external injury.

You choose the suitable instruments, and exert your mechanical skill in packing the material into place.

The hammer, the mallet, the chisel, the punch, or the spatula, may, any or all of them, be brought into requisition. Success or failure will be the test of mechanical ability, or the want of it. Take up any other point in dental practice, and the same observations hold good.

If then you can find no part of dentistry which is not mechanical, why, in the name of common sense is the phrase applied only to a single branch? Why not follow the nomenclature of medicine, and divide the science into Practical Dentistry and Theoretical Dentistry?

If Dental Theory can enable you to trace the pain of a rheumatic shoulder to a carious tooth, Dental Practice will remove the tooth, or apply some other remedy. We should then have Theory and Practice of Dentistry, just as we now have the Theory and Practice of Medicine.

The two sister sciences might then go hand in hand in their works of mercy. The elder not fearing that her dignity was in danger of being soiled by too close association with the "vulgar mechanic?"

I have asked the question how such an unnatural division of the duties of our profession has crept into the vocabulary of dental literature, and been tolerated by the profession.

Perhaps we may find its solution in a condition of things which existed in the past, more than at the present day: I allude to the two principle sources from whence its members were formerly recruited: the mechanical work shop, and the medical profession. On the one hand, then as now, our shops all over the land were filled with intelligent, enterprising men, all striving, not only to acquire property, but to raise themselves to a higher level in the social scale.

Such a man, when he went to the country doctor, and submitted himself to the agony of the turnkey, could not well avoid asking himself, whether he could not do the thing just as well, or better himself.

It is not strange that good mechanics should seek to become dentists.

Already possessed of mechanical tact, and the use of tools, it is not difficult to acquire sufficient theoretical knowledge to become successful operators and useful men.

On the other hand, the medical profession had been overstocked.

All could not succeed alike—some did not succeed at all.

They had taken the honors—had been "dubbed doctor," but the people were slow to trust their precious health in their keeping. It was not strange, therefore, that men occupying such a position, should consent to take one step down, and reap a living from fields they had not sown. They were brought in contact, and contrast, with dentists who had graduated from the mechanic shop. They needed his mechanical abilities, but feared the loss of dignity if placed upon his level. These men therefore, prefixed the adjective to their title, and came before the public as *Surgeon Dentists.*

They had well and truly laid the foundation of a good dental education. They were possessed of just those qualities in which the other class was most lacking; and they were deficient in those qualifications which rendered the mechanic so apt a scholar in the art and science to which he had aspired. Many of the doctors, it is true, before offering their services as dentists, took the pains to acquire this mechanical adaptability, and prepare themselves for practice, but others did not. They learned to perform such portion of their duties as must of necessity be performed in the presence of the patient.

Beyond this, their work was sent to the mechanic shop, or to a workman in the back room, who could work in metals—carve and bake their teeth—get up their models—cast their dies—swage their plates, and in short, could do just those things which they themselves could not do.

The one saw the patient, extracted the teeth, took the impression, and received the credit. The other possessed the skill, executed the work, was blamed for any want of adaptation, and received generally a small pittance of the profits. The one styled himself a Surgeon Dentist. The other was called a Mechanical Dentist; and the work which he could execute without ever seeing the face of the person for whom it was designed, was called *Mechanical Dentistry!*

The artistic element was necessarily ignored. The me chanic made a beautiful set of teeth; beautiful for the show case; all regular, well arranged, neatly soldered, highly polished, but when placed in the mouth, where was the expression? They might or might not harmonize with the features.

The winning smile, perhaps has become a silly grin. The grace which had always electrified, is no longer there. Some little twist, perhaps of one tooth merely, would make all right. The patient can not tell, her friends do not know the cause, but they do know that she is not quite herself again.

Such dentistry was truly mechanical—nothing else, and if it was such you wish me to write upon, I should feel myself constrained to decline the honor. To excel in this department, the mind that conceives, must be able to guide the hand that executes the work. Without this, as well might the sculptor design a statue, and then employ the mason to model the plastic clay; or the artist conceive an angelic picture, and then employ a painter to lay on the colors. In either case the soul, the life, the expression would be wanting. Just so in artificial dentistry; it must harmonize with the living features; and the faculties required to excel in this department, are the same as those brought into play by the sculptor, when his mind conceives and his hand brings forth the expression of pain, anger, remorse, despair, revenge, or other of the passions.

I decline therefore to recognize the phrase in this sense, and by Mechanical Dentistry, I shall understand as meaning *all* that is mechanical in dentistry. In thus contending for the proper use of terms, and at the same time admitting our profession to be essentially mechanical, I would not abate one iota of the learning requisite to practice it with intelligence; nor, in my opinion, do I thereby sacrifice any of its dignity.

By adopting this interpretation, perhaps you think I am taking a broad subject for this essay.

Do not be alarmed. I only take a wide range from which to choose a topic. I shall, therefore, in what I now say, confine myself to Artificial Dentures. Without going back into antiquity to search out when the first artificial tooth was set, it will be sufficient to say that at no very distant day, even within my own recollection, Artificial Dentures were made of bone or ivory, or the sea horse tooth. The blocks were carved into shape of teeth, and fastened by means of ligatures, or wires twisted round the necks of the adjoining teeth. When whole sets were inserted, they were retained in place by spiral springs, playing outside the teeth within the cheeks.

I have now in my possession, such a set, made in Paris for one of our foreign Ministers, as late as the year 1840. It was nicely carved into the shape of gums. The molars and bi-cuspids were carved from the same piece, but the incisors and cuspidati were human teeth fastened upon the blocks, precisely as we now insert pivot teeth upon the fangs of decayed teeth. The gums, I was informed, were colored, and the whole no doubt, presented an artistic appearance when new. They had been in use about two years. When brought to my office for repairs, the color of the gums had disappeared. One of the human teeth had decayed or dropped out.

The bone had softened, and was covered with a coat of slime.

In one place it had parted, and was held together with strings.

Of course, it was passed the possibility of repair, but I mention it to show the point from which the present advance has been made, within the recollection of many members of the profession, who are still in practice.

The first advance from this, which came to my notice, was the swaging up of gold plates to fit the alveolar ridge. They were first made very narrow, just wide enough to receive the base of the teeth, and upon this, the teeth, either human, animal, or ivory carved into shape, were riveted. These were more durable and cleanly, but it is needless to say they often required repairs, and must have been almost as much annoyance as benefit to the wearers.

Next came the French porcelain tooth. In shape it resembled half a bean, flat upon the back side, with a longitudinal channel to receive a wire standard, and a few platina points to fold over and be soldered to it.

This was a great advance. True, they were all of one shape and color, but they could be ground into any shape desired, and color was of little moment when offset by the advantage of durability. This seems to me to have been the starting point in modern dentistry. It pointed out the material which could be used for all time to come. It opened a field for experiment which has been occupied by a vast number of persons with untiring energy and wonderful skill and perseverance.

Next came the plain plate tooth with platina pins in the back, to which a metallic standard could be firmly soldered. Then came the pivot tooth, well shaped, and to a very good degree imitating the natural organ. I believe Mr. Samuel Stockton, a dentist of Philadelphia, was the first in this country to attain these results. At least, he was the first to bring his products into market, and place them within the reach of his professional brethren. The gum-tooth made its appearance somewhat later.

I hope I shall not be accused of egotism, if in this connection, I mention that the first one I ever saw, or heard of, was made in the office of Hayes & Harvey, and was inserted in the mouth of a lady who no longer needs the aid of anything artificial. It was but a few weeks later, however, when Mr. Stockton, in one of his annual visits to this city, took from his vest pocket two or three gum teeth.

The gum part was properly shaped and *painted red.* Not by any means, however, so good an imitation of the natural gum, as the enamel, colored with precipitate of gold, which had been prepared by myself and baked in our own furnace.

From this time the improvement in gold plate work was constant and rapid. At first the gum part of the tooth was ground to fit the plate like an ordinary plate tooth.

At a later time, the whole plate was encircled with a band of gold, into which the end of the tooth was placed as into a socket.

The plate also assumed broader dimensions, covering a larger portion of the roof of the mouth, thus giving a firmer hold of the plate by atmospheric pressure. The plate also was greatly strengthed by soldering together the edges of the standards up to a point opposite the point of the gums, and finally by filling all the crevices with a cement, they were rendered impervious to the collection of food or other substances, which might be taken into the mouth, and were thus rendered cleanly and wholesome. The limits of this essay will not permit me to enter minutely into details of the manner and manipulations of putting up this kind of work, but as the work itself seems rapidly to be passing into history, it may be well to state a few of the leading processes, which have proved most successful in my hands. We will take an upper plate for example.

The impression is taken with plaster of Paris in an impression cup, so shaped that the plaster when set, can not get loosened by the subsequent processes. This impression is baked or dried so as to expel all the moisture, but must not be exposed to a heat sufficient to decompose or soften the plaster. This impression, thus dried, is used as the mould in which the die is cast. This die is composed of tin and antimony, in proportions to render the metal hard but not brittle, and which will not shrink to any appreciable extent in cooling. The female die is made of pure tin, and the plate swaged up in the usual manner.

Wax is then placed upon the alveolar ridge of the plate, and carved into the shape of teeth; then placed in the mouth, and such alterations made, in shape, length, &c., as may be deemed desirable. A line is then drawn around the face of this wax, dividing it into two portions, one of which represents the teeth and gums, the other, the gold band into which they are to be placed.

The whole of the wax representing the teeth and gums is then cut away leaving that portion only which is to be replaced by the gold band.

A new mould is then taken in plaster from this part, a die is cast, and a strip of gold is swaged into the exact shape. This is soldered to the edge of the plate, which is then ready for the final process of adjusting the teeth. To do this, wax is again placed upon the plate, carved into the shape of teeth as before, the length, shape and position, properly adjusted, and the bite or articulation with the under teeth obtained, leaving indentations in the edge of the wax. The plate being removed from the mouth, the wax representing one of the central incisors is first cut out. In place of this a tooth is fitted, backed, and cemented into place. Then its mate, and so on, alternately one on each side till the whole are adjusted. The plate is then ready to be enveloped in plaster and sand for the soldering process. But before this is done, a slight dove-tail or notch is cut in each posterior angle of the plate, into which is placed a brace of iron wire, flattened at the ends so as to be chisel-shaped.

This is to prevent what has been known as the warping of plates, a trouble arising from the fact that the plate swells and shrinks more in the process of soldering than the teeth do by which it is surrounded; and unless thus braced is sure to be drawn into a more concave form than the die upon which it was stamped.

The whole is then enveloped in sand and plaster, held in place by a wrapper of wire cloth, barely exposing the parts to be soldered. The remaining processes are too familiar to you all to require repetition. I will only say in addition, therefore, that by soldering together the edges of the standards, up to the points of the gums, the plate is greatly stiffened, and all the crevices under and between the teeth, are converted, as it were, into cup-shaped cavities, and when filled with any suitable cement, (colored wax or paraphine,) are rendered wholly impervious to the secretions of the mouth, and remain so unless placed in boiling water; and then the cement is easily replaced, rendering this style of work both sweet and cleanly.

It would seem that little more could be expected of these materials, either in strength, beauty, or durability. It is not strange, therefore, that minds which had been on the alert for improvements, should stray into other fields of investigation and experiment. You all recollect the thrill of excitement produced by the announcement of the continuous gum work. Dr. Hunter sent his samples to the World's Fair, in London. Dr. Allen obtained his patent for substantially the same thing. The suit, the defense, the trial, all passed before the eye of the profession, as an absorbing panorama. That system of work not only grew into favor like the gourd of Jonah, but it faded also in like manner. Hardly had the rights of the parties been legally settled, before so much of the work was coming back for repair, and for other substitutes, that dentists were glad to clear themselves from its use as fast as possible. And yet, in the hands of proper artists it was capable of being made reasonably strong, and more artistic and beautiful than any other work which had gone before, or which has followed in its place. The porcelain work of Dr. Loomis came too soon upon the heels of this failure, to produce much excitement, or to induce many to experiment with its manipulation. The idea was, to carve the plate and teeth all from one piece of tooth body, and when baked, to have it fit the mouth for which it had been designed. When you consider that all tooth-body shrinks at least one eighth, in all dimensions, while undergoing the hardening process, you may readily judge of the difficulties to be encountered. Dr. Loomis did, however, by long practice, succeed in establishing some rules, and processes, by which he attained a reasonable degree of certainty. Some few others also, who went into that speciality, met with equally good success. I have seen many peices worn with great satisfaction; light, strong and beautiful. No possibility of the collection or absorption of the secretions of the mouth; easily washed as a china plate; and what seems strange, they are no more subject to accidents and fracture, if as much so, as most other styles of work. In comparison with gold, however, they are thick and clumsy in the mouth. They can not be made less than one sixteenth of an inch in thickness in the roof, and often are left much thicker.

Patients will get accustomed to anything that they suppose *must* be endured, but this is no excuse for the dentist, whose business it is to take all these things into account, and select the best material for his patient which is placed at his command. In point of chronological order, I now come to the epoch of the Vulcanite Base.

No change was ever more sudden, no revolution ever more perfect.

There seems to have been a wonderful conjunction of circumstances, all tending to enforce universal acceptance. It is cheap—the material costing nothing, comparatively. The processes are all simple, the farmer or the seamstress of last month, being the dentist of this in successful practice, with students in training, ready to be graduated, the next, or at the farthest, the month after. The dentist of long standing, and acquired fame, is bewildered. He sees his patients slipping through his fingers, and he too, in utter terror, adds his feeble cry to the accumulating host, "Better than gold!" "Better than gold!!" "Better than gold!!!" No wonder the monopolists have been laughing in their sleeves, while the rejected gold has been finding its way into their pockets. No wonder they are preparing new ones, broad and deep, and a pair of them at that; one for the use of the vulcanite, the other for the privilege of making it into plates. These pockets must all be filled from the pickings of this honorable profession. The man who has cried "better than gold!" can not well recede. For consistency's sake, if for nothing else, he must keep up the cry till his last pin-feather has been plucked.

I shall not discuss the relative merits of rubber as compared with former modes of practice.

It is enough for me to know that it has become a fixture in the Dental Laboratory, and can never be wholly expelled. It must have some advantages, or else it could not have sprung into so general use.

It has certainly extended the benefits of dentistry to a large class who could not formerly encounter the expense, and it is quite evident, I think, that much larger sums have been paid to the profession, in the aggregate, for artificial teeth, than would have been received, had their work been confined to any, or all the former modes of practice.

While various plans have been proposed for mounting teeth on rubber plate, it seems to me there is yet large room for progress. The moulded block work which has been so generally adopted, has introduced a stiff artificial uniformity, which is any thing but agreeable or artistic. Scarcely one set in fifty that does not reveal itself the first word that is uttered, or the moment the lips are parted. Not one set in a hundred, where the dentist, if he aspires to harmonize the features, would not like to give one tooth a twist here, another there, and so on. But this he can not do, and he seldom dismisses his patient, feeling that he has fully reproduced that harmony of form which nature had originally bestowed.

Finally, gentlemen, I would advise you all to keep well posted in all the departments of artificial dentistry. These spasmodic efforts at something new, are fostered more by the jealous fear that some one else will gain a march, than by any real conviction of their superior advantages; and that practitioner who carefully examines all the new projects which are brought forward, but adopts none till he is fully satisfied that they will result in benefit to his patients, not only preserves his own dignity and self-respect, but will be most likely in the end to secure the confidence and esteem of those to whom his services have been dedicated.

